# Structural View of a Non Pfam Singleton and Crystal Packing Analysis

**DOI:** 10.1371/journal.pone.0031673

**Published:** 2012-02-20

**Authors:** Chongyun Cheng, Neil Shaw, Xuejun Zhang, Min Zhang, Wei Ding, Bi-Cheng Wang, Zhi-Jie Liu

**Affiliations:** 1 National Laboratory of Biomacromolecules, Institute of Biophysics, Chinese Academy of Sciences, Beijing, China; 2 College of Stem Cell and Molecular Clinical Medicine, Kunming Medical University, Kunming, China; 3 Department of Immunology, Tianjin Medical University, Tianjin, China; 4 School of Life Sciences, Anhui University, Hefei, Anhui, China; 5 Department of Biochemistry and Molecular Biology, University of Georgia, Athens, Georgia, United States of America; University of Oulu, Finland

## Abstract

**Background:**

Comparative genomic analysis has revealed that in each genome a large number of open reading frames have no homologues in other species. Such singleton genes have attracted the attention of biochemists and structural biologists as a potential untapped source of new folds. Cthe_2751 is a 15.8 kDa singleton from an anaerobic, hyperthermophile *Clostridium thermocellum*. To gain insights into the architecture of the protein and obtain clues about its function, we decided to solve the structure of Cthe_2751.

**Results:**

The protein crystallized in 4 different space groups that diffracted X-rays to 2.37 Å (P3_1_21), 2.17 Å (P2_1_2_1_2_1_), 3.01 Å (P4_1_22), and 2.03 Å (C222_1_) resolution, respectively. Crystal packing analysis revealed that the 3-D packing of Cthe_2751 dimers in P4_1_22 and C222_1_ is similar with only a rotational difference of 2.69° around the C axes. A new method developed to quantify the differences in packing of dimers in crystals from different space groups corroborated the findings of crystal packing analysis. Cthe_2751 is an all α-helical protein with a central hydrophobic core providing thermal stability via π:cation and π: π interactions. A ProFunc analysis retrieved a very low match with a splicing endonuclease, suggesting a role for the protein in the processing of nucleic acids.

**Conclusions:**

Non-Pfam singleton Cthe_2751 folds into a known all α-helical fold. The structure has increased sequence coverage of non-Pfam proteins such that more protein sequences can be amenable to modelling. Our work on crystal packing analysis provides a new method to analyze dimers of the protein crystallized in different space groups. The utility of such an analysis can be expanded to oligomeric structures of other proteins, especially receptors and signaling molecules, many of which are known to function as oligomers.

## Introduction

One of the perplexing outcomes of sequencing of a number of genomes is the discovery of a large set of open reading frames (ORFs) in each genome that have no homologues in other species. Such ORFs, referred to as singletons or ORFans, have a codon usage pattern similar to those seen for other proteins, suggesting that these ORFs encode and express proteins [1]. Recently, singletons have attracted the attention of evolutionary biologists, biochemists and structural biologists regarding their origin, functional significance and the possibility that they may carry a relatively untapped source of new folds. Several hypotheses have been put forward to explain the lack of sequence identity and the origin of singletons; the most common explanation being that singletons are fast-evolving genes that have accumulated substitutions to such an extent that the sequence is no longer identical to the parent or any other known sequence [2]. Theoretical analysis of lineage specific genes involved in adaptation of a species to a particular environment seem to suggest that these genes are fast evolving since they have a “substrate” to act on and therefore a number of lineage-specific singletons have been postulated to play a role and confer an adaptive advantage on a particular species [3]. In contrast to this hypothesis, studies on the *Drosophila* genome show that singletons in *Drosophila* have similar rates of evolution as non-singletons and therefore accumulation of mutations may not be the only method for the origin of *Drosophila* singletons. Instead the singletons seem to have largely originated by *de novo* synthesis from non-coding regions like intergenic sequences [4]. In addition, insertion of transposon elements has resulted in completely new coding sequences. Such retro genes of viral origin have also been found to encode new proteins in primates [1], humans [5] and microbes [6]. The origin of singletons in mouse is partially attributed to frameshift mutations resulting in novel open reading frames [7]. Similarly, in *Saccharomyces*, ORFan domains are found at the C-termini of proteins and seem to have originated from frameshift mutations [8]. However, a majority of *Saccharomyces* ORFan domains are a result of *de novo* synthesis from non-coding DNA [8]. Thus, it seems that although different species might prefer one mechanism over another for the generation of singletons, they might still be using all of these methods – a faster rate of mutation, *de novo* synthesis from non-coding DNA, lateral gene transfers via transposons and frameshift mutations – to produce singletons. One question that arises then is – are singletons merely aberrations of biological processes or do they play a role in the survival and propagation of organisms? Attempts have been made to address this question and there is strong evidence now that singletons express protein. For instance, in a genome-wide study on *Halobacterium*, the authors could detect mRNA for 30 out of 39 paralogous singletons representing 13 out of 14 families identified in *Halobacterium* [9]. Similarly, singleton genes involved in immune response, oxygen stress, flight and circadian rhythm could be detected in the cDNA of *Drosophila yakuba* suggesting singletons are expressed as legitimate proteins. A mutation in the singleton *fln* gene that encodes protein for a thick filament in flight muscle results in a viable but flightless fly [10]; a mutation in the circadian rhythm *to* gene produces a rhythm defective fly [11]. Interestingly, all these functions of singletons are expected to play a role in the fly's response to specific ecological or environmental challenges. Although these examples underscore the fact that singletons are expressed as proteins and play a functional role, a vast majority of singletons yet have unknown functions.

One way to gain functional insights is to solve the 3-dimensional structure of the protein and compare it with structures with known function deposited in PDB [12]. This method is more sensitive than the primary sequence match because structure is more conserved than sequence. For example, the protein MJ0882 (GI #1499712) from *M. jannaschii* was annotated as a hypothetical protein with unknown function [13]. The primary sequence provided no clues about the function. When the crystal structure of the protein was solved, it revealed a methyl-transferase fold. The protein was subsequently assayed for methyl-transferase activity and assigned a function. In many instances, clues about the function have been gained from ligands bound to the protein. These ligands can originate from the expression system or crystallization conditions [14]. Metal ions bound to proteins and the environment around the metal ion can often shed light on the function of the protein, which can then be validated experimentally. For example, a conserved zinc binding site for a protein YP_164873.1 from *Silicibacter* that was missed in primary sequence analysis due to low sequence identity to proteins with known function was revealed in the 3-D crystal structure. Comparison of the secondary structural elements and the Zn-binding residues with 3-keto- 5-aminohexamoate cleavage protein helped assign a function to the protein [14]. Similarly, fortuitous binding of phosphate, ADP, ATP, NADP, NAD, SAM, fatty acids, DNA, etc coupled with information about the fold, has helped decipher functions for proteins previously annotated with unknown function [15].

Cthe_2751_is a 15.8 kDa singleton from an anaerobic, hyperthermophile *Clostridium thermocellum*, with an unknown function. The primary sequence of Cthe_2751 displays no identity to any protein with known function and does not provide any clue to its functions. Therefore, we decided to solve the crystal structure of the protein to gain insights into the architecture of the protein and obtain clues about its function. The structure solved to 2.17 Å resolution by Se-SAD reveals an all α-helix topology. A crystal packing analysis of the different crystal forms of Cthe_2751 was performed to investigate the molecular packing preferences of the different space groups. Potential functions of the protein based on motifs observed in the structure are discussed.

## Results

### Primary sequence analysis

**Figure 1:**
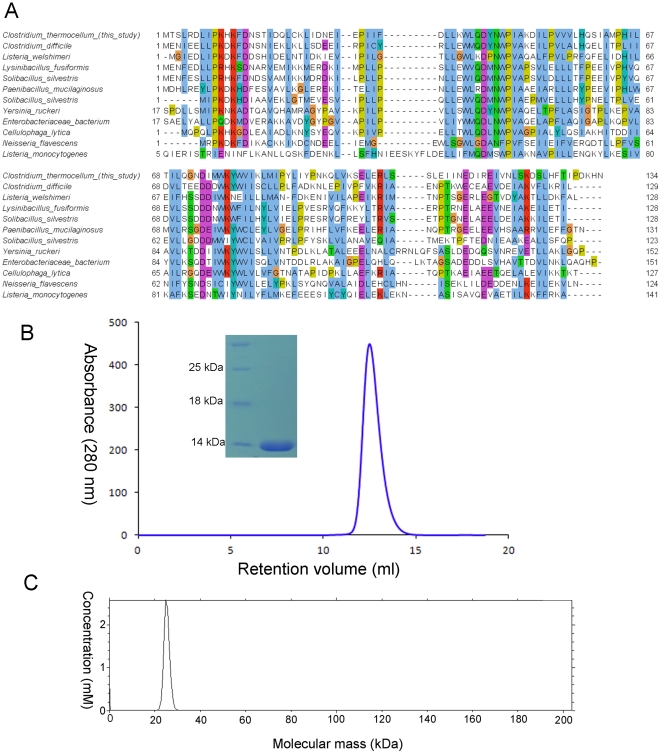
Characterization of Cthe_2751 (A) Sequence alignment of Cthe_2751 homologues. Only the top 11 matches with Cthe_2751 amino acids 1–134 are shown. Conservation is colored according to ClustalW convention. (B) Size exclusion profile of Cthe_2751 run on Hi Load 10/300 Superdex G75 gel filtration column equilibrated with 20 mM Tris-HCl, 100 mM NaCl, pH 8.0, reveals that the protein exists as a dimer in solution. SDS-PAGE picture (inset) showing the purity of Cthe_2751 before crystallization. (C) Sedimentation velocity experiments performed using an analytical ultracentrifuge suggested that Cthe_2751 forms a dimer in solution. The curve was generated using Sedfit software.

A PSI-BLAST [16] search of the non-redundant protein sequences deposited in GenBank [17] failed to retrieve any similar sequence with known function (Figure 1). A Pfam search using the primary amino acid sequence of Cthe_2751 revealed that the sequence could not be assigned to any of the known protein families. Interestingly, Cthe_2751 is produced only by *Clostridium thermocellum*. The closest homologue from *Clostridium difficile* shares less than 45% sequence identity with Cthe_2751. Homologous sequences from other species share 31% or less identity. Therefore, based on primary sequence analysis, Cthe_2751 is a non-Pfam singleton with an unknown function.

### Overall structure

**Figure 2:**
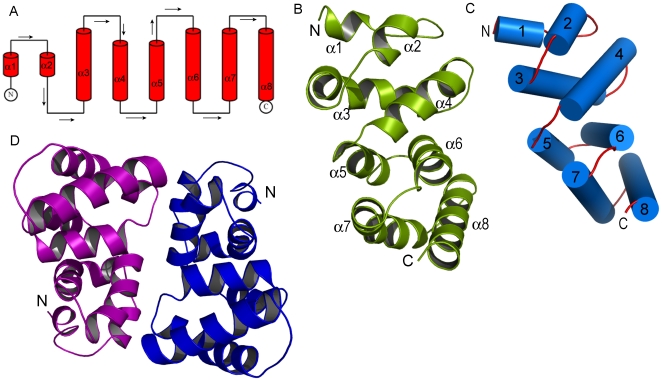
Structure of singleton Cthe_2751. (A) Topology diagram of the structure. (B) Cartoon representation of Cthe_2751 with helices shown as cylinders in (C) to depict the contour, pairing and stacking. (D) Cartoon representation of a dimer of Cthe_2751.

Cthe_2751 could be purified to homogeneity using Ni-affinity and gel filtration chromatographies (Figure 1B and 1C). The structure was solved by the Se-SAD method. The 2.17 Å crystal structure of Cthe_2751 in space group P2_1_2_1_2_1_ consists of α-helices and loops with no β-strands (Figure 2A, 2B and 2C). Each monomer is made up of 8 α-helices arranged in a spiral pattern around a vertical axis that runs through the centre of the protein. The turns in the spiral are facilitated by 4 β and 1 γ turn motifs. The helices are arranged in anti-parallel pairs. The α1/α2 pair of helices is seen stacked above the α3/α4 pair and forming a module. Similarly, the α5/α6 pair is seen stacked above the α7/α8 pair and forming the second module. This module is rotated by approximately 30° along the vertical axis of the spiral with respect to the first module (Figure 2B and 2C). The modules are held together *via* numerous hydrophobic interactions involving aromatic residues.

### Crystallographic packing analysis

**Figure 3:**
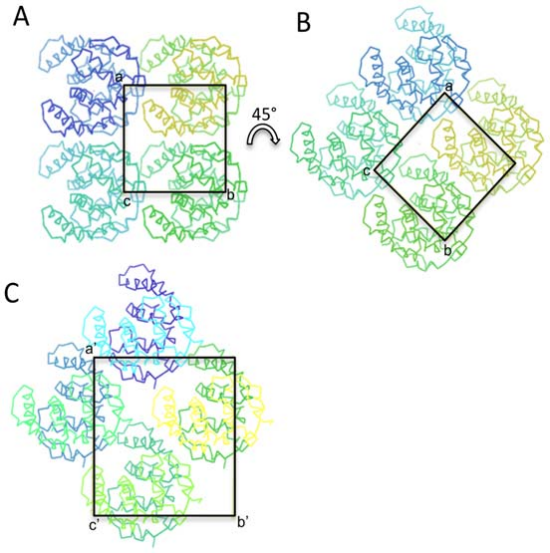
Two dimensional projection (along C axis direction) of 2-fold symmetry related molecules for space group P4_1_22 and C222_1_. The transformation between space groups P4_1_22 and C222_1_ is illustrated. (A) The projection of Cthe_2751 monomer (dark blue) and 7 symmetry related molecules along C axis. The homodimer of Cthe_2751 (dark and light blue molecules) is related by crystallographic 2-fold 2(x 0 0). Please note that 4_1_ screw symmetry related molecules are not shown for the sake of only displaying the transformation between P4_1_22 and C222_1_ space groups; (B) In order to illustrate the transformation between P4_1_22 and C222_1_ space groups, Figure 3A is rotated 45 degrees clockwise around 4_1_ axis; (C) The projection of Cthe_2751 dimer along C axis. The 4 Cthe_2751 dimers in C222_1_ space group have almost the same orientation as that of the 8 Cthe_2751 monomers (or 4 dimers) in the 45° rotated P4_1_22 unit cell.

**Figure 4:**
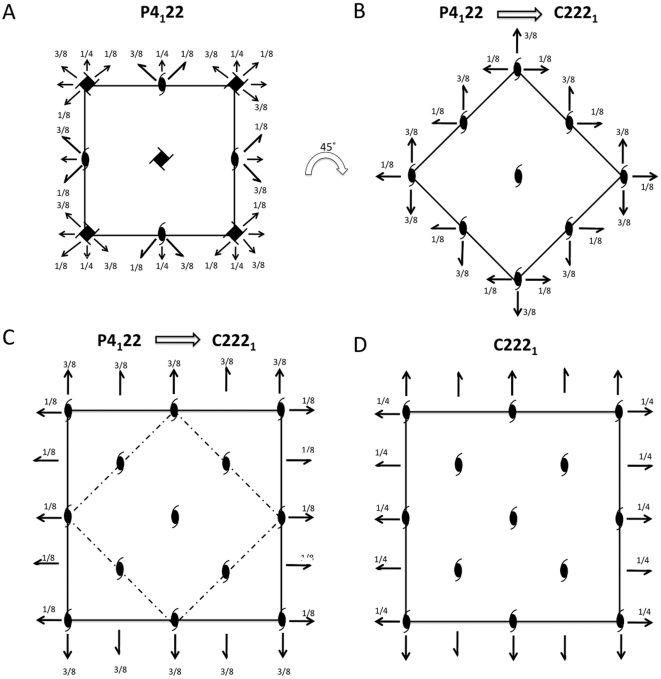
The transformation between space groups P4_1_22 and C222_1_. (A) The projection of symmetry elements in space group P4_1_22 along 4_1_ axis; (B) Degeneration of P4_1_22: 4_1_ axis are transformed into 2_1_ axis, while the 2 fold axis, generated by 4_1_ axis, disappear too. The cell is rotated clockwise by 45° around 4_1_ axis; (C) The cell parameter a′ and b′ in C222_1_ space group take the diagonal direction along **a+b** and **a−b** of space group P4_1_22, respectively, and forms new unit cell; (D) In order to follow the international conversion of space group C222_1_, the origin is translated 3/8 cell length along c axis.

Pure Cthe_2751 eluted as a dimer when subjected to size exclusion chromatography. Further, sedimentation velocity experiments using an analytical ultracentrifuge [1]suggested that pure Cthe_2751 was homogenous and dimeric. Therefore, Cthe_2751 probably exists as a dimer in solution (Figure 1B and 1C). To find out whether the protein crystallized as a dimer and obtain information on the nature of the interface, we performed crystal packing analysis. The wild-type Cthe_2751 crystallized into 3 different crystal forms belonging to space groups P4_1_22, C222_1_ and P2_1_2_1_2_1_, respectively. The selenium labelled protein crystallized into P3_1_21 space group which has 1 molecule of Cthe_2751 plus a small fragmented helix in the asymmetric unit. The extra helix seems to have originated by proteolysis during the crystallization incubation process. In the three space groups of wild-type protein, the minimum crystal packing unit is a dimer of Cthe_2751 (Figure 2D). In crystal forms C222_1_ and P2_1_2_1_2_1_, there is one dimer per asymmetric unit. Although the crystal form P4_1_22 has only one molecule in the asymmetric unit, a careful inspection of the asymmetric unit revealed the presence of an identical dimer of Cthe1904 as seen in other 2 space groups, with the monomers within the dimer related by a crystallographic 2-fold symmetry axis. A detailed analysis of the crystallographic packing of different crystal forms showed that there is very subtle difference between the crystal packing of space groups P4_1_22 and C222_1_. The unit cell parameters of these two space groups are: a = b = 37.51 Å, c = 169.75 Å (P4_1_22); a = 52.04 Å, b = 55.95, c = 170.83 Å (C222_1_). Theoretically, when the space group P4_1_22 transforms to a lower symmetry C222_1_ space group, the 4_1_ screw axis degenerates to a 2_1_ screw axis with a concomitant disappearance of the 2-fold axes in **a** and **b** directions. The **a′** and **b′** in C222_1_ space group takes the diagonal direction along **a+b** and **a-b** in P4_1_22 unit cell, respectively, as shown in Figures 3 and 4. The diagonal length | **a**+**b** | = 53.05 Å in P4_1_22 unit cell agrees well with the average length of **a′** and **b′** (53.99 Å) of space group C222_1_. In the transformation from P4_1_22 to C222_1_, the Cthe_2751 dimers rotate only 2.69° around the C axes.

As for the crystal form P2_1_2_1_2_1_, the packing arrangement of the dimers clearly deviates from those found in space groups C222_1_ and P4_1_22. There is a 60.9° orientation difference between the corresponding dimer in P2_1_2_1_2_1_ and that in other two space groups (C222_1_ and P4_1_22). To find out if there is a difference in the packing arrangement of the dimers in the 3 crystal forms and to quantify it, a computer program was compiled and a calculation was performed to analyze the inter-dimer distances of 2 neighbouring dimers for all 3 space groups. Specifically, the inter-dimer distance between the Cα atoms of each residue with that of the residues in the closest neighbouring dimer was computed. Theoretically, if the dimers share similar packing arrangements 3-dimensionally in different space groups, the corresponding inter-molecular distances between neighbouring dimers should show small r.m.s. deviations and good correlations. The computed results are listed in Table 1. As expected, the inter-dimer distance between 2 closest dimers in space groups C222_1_ and P4_1_22 is relatively similar when compared to that of the distance between dimers of space groups P2_1_2_1_2_1_ and C222_1_ or P2_1_2_1_2_1_ and P4_1_22, where there is almost no recognizable co-relationship. This result further supports the inferences of crystal packing analysis where we saw that the dimers rotate less than 3° along the C axes during the transformation from P4_1_22 to C222_1_ resulting in only a minor change in crystal packing.

**Table 1:** Statistics of inter-dimer distance between 2 neighboring dimers in the 3 space groups.

Rmsd*(Å)/corr&	P2_1_2_1_2_1_	P4_1_22	C222_1_
P2_1_2_1_2_1_	-	17.28/0.40	17.10/0.40
P4_1_22	17.28/0.40	-	0.69/0.99
C222_1_	17.10/0.40	0.69/0.99	-

*: RMSD is calculated based on the following equation:
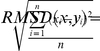
.

& : Pearson correlation coefficient is calculated based on the following equation:
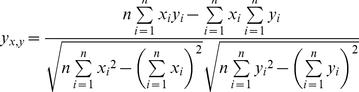
Where x and y are the corresponding inter-dimer distances, n is the number of atomic pairs.

### Dimer interface

**Figure 5:**
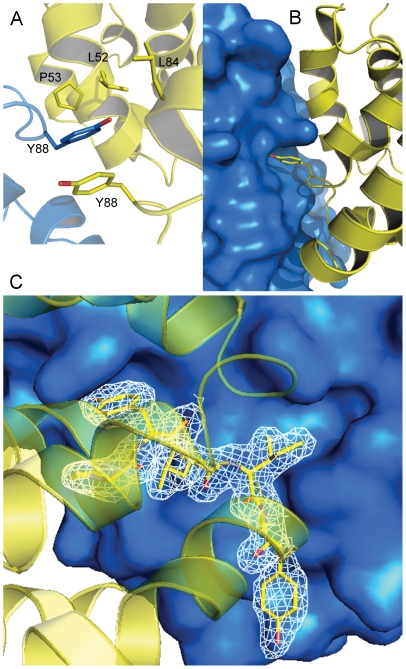
Dimer interface. (A) The side chain of Tyr88 of chain A protrudes into a concave cavity formed by Leu52, Pro53, Leu84 and Tyr88 of chain B. (B) Chain A shown in surface representation, while chain B is depicted as a cartoon. (C) Representative 2*Fo-Fc* electron density for some of the residues at the dimer interface contoured at 1.0 σ.

Cthe_2751 crystallized as a dimer in 3 different crystal forms. Superimposition of the dimers crystallized in different space groups revealed no obvious differences in the position of the Cα atoms suggesting an identical mode of dimerization in all the 3 crystal forms. We performed Protein Interfaces Surfaces and Assemblies (PISA) [18] analysis to identify the dimer interface. The analysis revealed that dimerization occurs *via* a large area that spans 904 Å^2^ (12.8%) of the surface area per monomer. Formation of the interface results in a gain of 8.6 kcal/mol of free energy of solvation (Δ^i^G). This interface scored 1.000 in Complexation (complex formation) Significance Score (CSS). CSS ranges from 0 to 1 as the relevance of the interface to complex formation increases. Further, PISA identified 6 intermolecular hydrogen bonds holding the monomers together within a dimer (Table 2). Interestingly, the aromatic ring of Tyr88 from one monomer protrudes into a concave cavity formed by Leu52, Pro53, Leu84 and Tyr88 of another monomer, zipping the monomers together (Figure 5). The aromatic rings of the tyrosines stack against one another holding the monomers together within the dimer. In addition, numerous inter-molecular hydrogen bonds mediated by water molecules are observed stabilizing the dimer interface.

**Table 2:** Inter monomer hydrogen bonds identified by PISA analysis of the Cthe_2751 dimer.

No.	Chain A	Distance (Å)	Chain B
1	Tyr88OH	2.54	Leu84O
2	Tyr91N	3.63	Cys22SG
3	Lys94NZ	3.58	Asp50OD1
4	Cys22SG	3.74	Tyr91N
5	Asp50OD1	3.74	Lys94NZ
6	Leu84O	2.61	Tyr88OH

### Modeling studies

**Figure 6:**
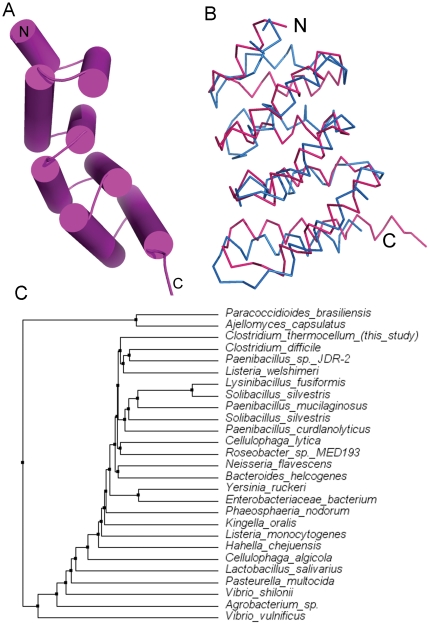
Modelling of Cthe_2751. (A) Cartoon of the model predicted by I-TASSER (B) Superposition of the Cα atoms of the predicted structure (magenta) over the experimental crystal structure (blue). (C) Average distance tree for Cthe_2751 constructed by the Jalview 2.6.1 Java alignment editor using BLOSUM62.

Singletons can serve as perfect probes for bench marking available protein structure prediction softwares. We modelled the structure of Cthe_2751 using 10 different web-based prediction programs that use a variety of methods like *ab initio* structure prediction, homology modeling, energy based structure prediction, threading, profile-profile alignment and HMM-based protein structure prediction (Table 3). The models predicted by these programs were compared with the experimental crystal structure of Cthe_2751. Parameters such as similarities in topology, lowest r.m.s.d., longest residue alignment length, average r.m.s.d. and average residue alignment length were chosen for the comparison. The best model closest to the experimental structure was predicted by I-TASSER (Table 3). Since Cthe_2751 has no homologous structure deposited in PDB, a modeling program like I-TASSER, which builds models by threading, was expected to give the best model. Although the r.m.s.d. of the superimposition of the Cα atoms on the experimental structure was 2.8 Å over a length of 108 out of 130 residues, visual inspection of the topology of the model revealed a remarkable similarity with the experimental structure (Figure 6 A and 6B). This exercise raises interesting possibilities of fairly accurate modeling of unique protein sequences having no homologues in PDB and with no Pfam assignments.

**Table 3:** Details of models built by different 3D structure prediction programs.

Program	Method	Number&Of Solutions	Lowest RMSD†	Longest aligned length	Average RMSD	Average aligned length	Reference
BHAGEERATH	Energy Based Structure Prediction Server	5	2.55	57	3.72	46	[35]
I-TASSER	threading methods	5	2.83	108	3.09	99	[36]
I-TASSER-ab	*Ab initio* structure prediction	10	2.34	90	3.26	69	
LOOPP	Multiple methods	5	2.40	81	3.00	63	
MUSTER	profile-profile alignment	9	2.52	85	3.18	73	[37]
PHYRE	the protein homology/analogy recognition engine	1	3.52	83	3.52	83	[38]
Pcons	Model Quality Assessment Program	10	2.25	90	3.05	66	[39]
(PS)2-v2	automatic homology modeling server	1	2.83	70	2.83	70	[40]
Robetta	Rosetta homology modeling and ab initio fragment assembly with Ginzu domain prediction	5	2.74	77	3.14	68	[41]
SAM_T08	HMM-based Protein Structure Prediction	2	3.77	106	3.80	100	[42]

† – Root mean square deviations in Å for corresponding Cα atoms of the best solution that were aligned with those of the crystal structure of Cthe_2751.

& - Number of Solutions is the number of models predicted by the software.

## Discussion

There is a general consensus that although the number of new ORFs is poised to grow further with sequencing of DNA from diverse sources, there may not be a concomitant large scale increase in the number of new protein folds. This is because, 2 proteins with low sequence identity can still share similar folds; implying fold is more conserved than sequence. What this means is that although the fold space could be limited, one would have to go through a large number of ORFs to cover this space. One strategy for hunting new folds is sifting through the largely untapped source of unique ORFs found in genomes of taxonomically distant organisms. Cthe_2751 is a singleton from a Gram positive, anaerobic, thermophilic bacterium found in soil. A phylogenetic tree of Cthe_2751 constructed from protein sequences obtained via a PSI-BLAST search [16] and alignment with ClustalW [19], clearly shows that Cthe_2751 is phylogenetically distant from other proteins (Figure 6C). Interestingly, proteins similar to Cthe_2751 are predominantly found in prokaryotes, with *Phaeosphaeria* and *Ajellomyces* being the exceptions. Cthe_2751 is more similar to hypothetical proteins from Gram positive bacteria like *Listeria*, *Paenibacillus*, *Lysinibacillus*, and *Solibacillus*. In general, Cthe_2751 homologues from Gram positive rod shaped bacteria cluster together. While a majority of homologues are from rod shaped bacteria, there are two notable exceptions – hypothetical proteins from *Nisseria* and *Kingella*, both of which are Gram negative cocci. Inspite of being phylogenetically distant, the structure of Cthe_2751 reveals that the sequence folds into a known all α-helical fold confirming the fact that unique sequences may not always give rise to new folds and that structure is more conserved than sequence [20].

**Figure 7:**
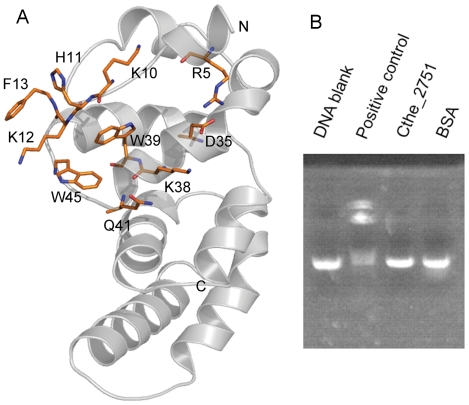
Functional analysis of Cthe_2751. (A) Cluster of aromatic and charged residues of Cthe_2751. N and C terminals are marked; Cthe_2751 is depicted as a cartoon, amino acids as sticks. (B) Nucleic acid binding ability of Cthe_2751 was tested in an EMSA assay. Cthe_2751 could not bind double stranded nucleic acids similar to bovine serum albumin (BSA) under the assay conditions. Lambda repressor protein was used as a positive control.

A web based server called ProFunc predicts function for a protein from its 3-D structure. We carried out a ProFunc analysis of the structure of Cthe_2751 to obtain clues about the function. Although no matching sequence motifs were found, a low sequence (25% identity) and E value (9.7) match with a splicing endonuclease from *Pyrobaculum* (PBD code 2ZYZ) was retrieved. A 3D functional template search module of ProFunc came up with a possible match with a RNA binding protein from *Mus musculus* (PDB code 1KEY). These clues suggested that the function of Cthe_2751 involved participation of nucleic acids. Next, we retrieved and analyzed the topology of protein structures known to bind nucleic acids and compared them with Cthe_2751. The CID domain of Pcf11 shows remarkable similarity in topology to Cthe_2751. The CID domain interacts with the CTD domain of RNA polymerase during processing of RNA [21]. Similarly, the C-terminal of *Pyrococcus woesei* transcription factor B (pwTFBc), which binds nucleic acids, has an all helical topology like Cthe_2751 [22]. In addition to the similarity in topology with proteins binding nucleic acids, inspection of the structure of Cthe_2751 reveals potential motifs for nucleic acid binding. For example, Cthe_2751 has a cluster of aromatic and charged residues similar to those seen around the RNA in the structure of *Archaeglobus fulgidus* splicing endonuclease [23]. Since Cthe_2751 has aromatic amino acids, lysines and arginines in a cluster on the surface, we decided to test if the protein could bind nucleic acids (Figure 7). Preliminary experiments reveal that Cthe_2751 could not bind double stranded DNA in an EMSA assay (Figure 7B) suggesting that either the binding specificities might be stringent or the function of Cthe_2751 may not have anything to do with nucleic acid binding. Further biochemical studies are warranted to unravel the function of Cthe_2751, which are currently underway.

### Conclusions

We have solved the 3-D structure of the non-Pfam singleton Cthe_2751 to 2.17 Å resolution by Se-SAD. The structure reveals an all α-helical topology similar to those observed for nucleic acid processing proteins. A mathematical calculation performed on the dimers of Cthe_2751 crystallized in different space groups corroborated the findings of the crystal packing analysis of molecules packed in different space groups. Such a method of analysis of packing of dimers can be extrapolated to the study of dimerization of proteins known to function as dimers under physiological conditions.

## Methods

### Cloning, expression and purification

The *Cthe_2751* gene containing 405 bases was sub-cloned into vector pMCSG7 to give an expression plasmid - pMCSG7-Cthe_2751 [24]. A number of single colonies were selected for small scale soluble protein expression screening. Interestingly, only 1 clone produced soluble protein. Sequencing results revealed a frameshift mutation in the clone expressing soluble protein. As a result, the C-terminal ^124^SLHFTIPDKHN^134^ region was changed to ^124^YLAFYY^130^ with a fortuitous stop codon ending the translation of the protein after Tyr130. Although amino acids Leu125 and Phe127 retain their positions, the overall effect of the base insertion is a 5 amino acid C-terminal truncation and mutagenesis of last 4 amino acids. Since the mutated amino acids are located at the C-terminal end, the effect on the structure due to the change in amino acids is likely to be minimal. Since this was the only clone that gave soluble protein, it was used for protein production. pMCSG7-Cthe_2751 was transformed into *E. coli* BL21 for protein production. Cells were grown at 37°C until the optical density of the culture reached OD_600 nm_ 0.8. The culture was induced by IPTG with a final concentration of 0.2 mM at 16°C for 20 h. Cells were harvested by centrifugation at 4000 rpm for 30 min, and lysed by sonication. After centrifugation at 30,670 g for 30 min, the supernatant was subjected to Ni-affinity chromatography. His-tagged protein was eluted using 1× PBS buffer containing 500 mM imidazole. After buffer exchange, the protein was subjected to a TEV treatment to remove the His-tag. Uncut protein and TEV were removed by a second round of Ni-affinity chromatography and the tag-less protein was loaded onto a Superdex G75 gel filtration column previously equilibrated with 20 mM Tris-HCl (pH8.0), 200 mM NaCl. The protein eluted as a single peak during size exclusion and was concentrated to 15 mg/ml, before setting up crystallization drops. Selenomethionine-labeled Cthe_2751 protein was produced from *E. Coli B834* by growing the cells in M9 medium supplemented with 0.5% glucose and 100 µg/ml selenomethionine at 37°C until OD_600_ reach 0.6. Labelled protein production was initiated by adding 0.2 mM IPTG and the cells were allowed to grow for further 30 h. The protein was purified as described earlier for the native protein.

### Crystallization

Crystallization experiments were performed by hanging drop vapor diffusion method by hand at 16°C. A total of 500 different conditions from commercially available sparse matrix screens were used for screening. Crystallization drops contained 1 µl protein solution mixed with 1 µl reservoir solution, and were equilibrated over 300 µl reservoir solution.

After 5 days of incubation, the protein crystallized. Crystals of selenomethionine labelled protein belonging to space group P3_1_21 grew in 0.2 M ammonium sulfate, 0.1 M Bis-Tris, pH 5.5, while crystals of native protein belonging to space groups, C222_1_, P4_1_22 and P2_1_2_1_2_1_ grew in 1.6 M magnesium sulfate, 0.1 M MES, pH 6.5, 30% PEG 8000, 0.2 M ammonium sulfate and 20% PEG 3350, 0.2 M magnesium nitrate, pH 5.8, respectively.

### Data collection, phasing, structure solution, and refinement

As expected, a WuBlast search of the PDB revealed that there were no structural homologues of Cthe_2751. Therefore, we prepared a selenomethionine derivative of the protein to obtain the phase information. Mass spectroscopy of the labelled protein suggested that all 4 methionines had been successfully replaced by selenomethionine (data not shown). Crystals were briefly soaked in a cryo solution containing the mother liquor supplemented with 10% glycerol before freezing them in liquid nitrogen prior to diffraction testing and data collection. The selenium labelled protein crystal diffraction data were collected at peak wavelength for selenium's anomalous scattering (0.9793 Å) at beamline 19-ID of Advanced Photon Source (APS), Argonne National Laboratory. Data for the other 3 crystal forms of wild-type protein were collected at either home lab or beam 17A at Photon Factory of KEK, Japan as shown in Table 4. All the diffraction raw images were indexed and scaled using HKL2000 [25]. The structure was solved by Se-SAD using program SHELX [26] and Phaser [27], in CCP4 Suite [28]. The model was automatically built with program Arp/Warp [29] in CCP4 Suite [28]. Except for the N-terminal methionine, anomalous signal of selenium for Met63, Met76 and Met85 could be detected. The experimental electron density map was of very good quality and other than Met1, which is disordered, most of the residues could be fitted unambiguously. The nearly complete model was used as a molecular replacement template in the subsequent structure determination of the three wild-type crystal structures using program Phaser [27], [30]. The models in different space groups were completed with several cycles of refinement including the use of TLS refinement method (Refmac [31] and Phenix_Refinement [32]) and manual fitting with Coot [33]. Details of data collection and refinement statistics are listed in Table 4. The quality of the final model was validated with MOLPROBITY [34].

**Table 4:** Data collection and refinement statistics.

Data collection	Se Derivative	Native 1	Native 2	Native 3
X ray source	19-ID, APS	MicroMax-007IP, IBP	17A, PF	17A, PF
PDB accession code		3UT8	3UT7	3UT4
Crystal to detector distance (mm)	362.87	180.42	308.73	299.58
Number of images	320	360	360	360
Oscillation width (°)	0.5	1	0.5	0.5
Wavelength(Å)	0.9796	1.5418	0.9800	0.9800
Space group	P3_1_21	P2_1_2_1_2_1_	P4_1_22	C222_1_
a,b,c (Å)	80.80, 80.80, 53.97	50.10, 63.88, 96.90	37.51, 37.51, 169.76	52.04, 55.95, 170.83
α,β,γ (**°**)	90.00, 90.00, 120.00	90.00, 90.00, 90.00	90.00, 90.00, 90.00	90.00, 90.00, 90.00
molecules	1	2	1	2
Resolution range(Å)	50.00–2.37(2.45–2.37)	50.00–2.17(2.25–2.17)	50.00–3.00(3.11–3.00)	50.00–2.03(2.10–2.03)
Rsym (%)	10.0 (47.0)	3.6(11.2)	4.8 (13.1)	8.1 (36.7)
Mean I/σI (I)	26.52 (4.16)	90.50 (30.98)	52.45 (15.04)	33.76 (5.51)
Completeness (%)	99.3 (94.8)	97.9 (92.4)	96.1(98.6)	99.7 (97.2)
Redundancy	7.6 (5.6)	13.1 (9.3)	10.5 (9.9)	6.6 (6.5)
**Refinement**				
Resolution (Å)	50.00–2.37	27.15–2.17	42.44–3.01	50.00–2.03
No. reflections	8055	16720	2562	15606
Rwork/Rfree (%)	19.23/23.43	22.28/22.66	21.19/23.75	22.65/27.05
No. atoms	1204	2298	1041	2245
Protein	141	256	127	256
Water	40	126	3	81
Mean B value (Å^2^)	35.40	35.87	41.80	25.15
**R.m.s deviations**				
Bond lengths (Å)	0.017	0.008	0.011	0.012
Bond angles (°)	1.258	1.058	1.553	1.093
**Ramachandran analysis**				
Favoured region (%)	99.26	99.60	95.16	98.02
Allowed region (%)	0.74	0.00	4.03	1.98
Outliers (%)	0.00	0.40	0.81	0.00

## References

[1] Toll-Riera M, Bosch N, Bellora N, Castelo R, Armengol L (2009). Origin of Primate Orphan Genes: A Comparative Genomics Approach.. Mol Biol Evol.

[2] Domazet-Loso T, Tautz D (2003). An Evolutionary Analysis of Orphan Genes in Drosophila.. Genom Res.

[3] Nielsen R, Bustamante C, Clark AG, Glanowski S, Sackton TB (2005). A Scan for Positively Selected Genes in the Genomes of Humans and Chimpanzees.. PLoS Biol.

[4] Zhou Q, Zhang G, Zhang Y, Xu S, Zhao R (2008). On the origin of new genes in Drosophila.. Genom Res.

[5] Marques AC, Dupanloup I, Vinckenbosch N, Reymond A, Kaessmann H (2005). Emergence of Young Human Genes after a Burst of Retroposition in Primates.. PLoS Biol.

[6] Yin Y, Fischer D (2006). On the origin of microbial ORFans: quantifying the strength of the evidence for viral lateral transfer.. BMC Evol Biol.

[7] Okamura K, Feuk L, Marques-Bonet T, Navarro A, Scherer SW (2006). Frequent appearance of novel protein-coding sequences by frameshift translation.. Genomics.

[8] Ekman D, Elofsson A (2009). Identifying and Quantifying Orphan Protein Sequences in Fungi.. J Mol Biol.

[9] Shmuely H, Dinitz E, Dahan I, Eichler J, Fischer D (2004). Poorly conserved ORFs in the genome of the archaea Halobacterium sp. NRC-1 correspond to expressed proteins.. Bioinformatics.

[10] Barton B, Ayer G, Heymann N, Maughan DW, Lehmann F-O (2005). Flight muscle properties and aerodynamic performance of Drosophila expressing a flightin transgene.. J Exp Biol.

[11] So WV, Sarov-Blat L, Kotarski CK, McDonald MJ, Allada R (2000). takeout, a novel Drosophila gene under circadian clock transcriptional regulation.. Mol Cell Biol.

[12] Watkins HA, Baker EN (2006). Structural and Functional Analysis of Rv3214 from Mycobacterium tuberculosis, a Protein with Conflicting Functional Annotations, Leads to Its Characterization as a Phosphatase.. J Bact.

[13] Shin DH, Hou J, Chandonia JM, Das D, Choi IG (2007). Structure-based inference of molecular functions of proteins of unknown function from Berkeley Structural Genomics Center.. J Struct Func Genom.

[14] Kumar A, Chiu H-J, Axelrod HL, Morse A, Elsliger M-A (2010). Ligands in PSI structures.. Acta Crystallogr F.

[15] Speers AE, Cravatt BF (2010). Ligands in crystal structures that aid in functional characterization.. Acta Crystallogr F.

[16] Altschul SF, Madden TL, Schaffer AA, Zhang J, Zhang Z (1997). Gapped BLAST and PSI-BLAST: a new generation of protein database search programs.. Nucleic Acids Res.

[17] Benson DA, Karsch-Mizrachi I, Lipman DJ, Ostell J, Sayers EW (2011). GenBank.. Nucleic Acids Res.

[18] Krissinel E, Henrick K (2007). Inference of Macromolecular Assemblies from Crystalline State.. J of Mol Biol.

[19] Brooksbank C, Cameron G, Thornton J (2005). The European Bioinformatics Institute's data resources: towards systems biology.. Nucleic Acids Res.

[20] Su J, Li Y, Shaw N, Zhou W, Zhang M (2010). Crystal structure of a novel non-Pfam protein PF2046 solved using low resolution B-factor sharpening and multi-crystal averaging methods.. Protein & Cell.

[21] Hollingworth D, Noble CG, Taylor IA, Ramos A (2006). RNA polymerase II CTD phosphopeptides compete with RNA for the interaction with Pcf11.. RNA.

[22] Kosa PF, Ghosh G, DeDecker BS, Sigler PB (1997). The 2.1 Å crystal structure of an archaeal preinitiation complex: TATA-box-binding protein/transcription factor (II)B core/TATA-box.. Proc Nat Acad Sci.

[23] Xue S, Calvin K, Li H (2006). RNA Recognition and Cleavage by a Splicing Endonuclease.. Science.

[24] Stols L, Gu M, Dieckman L, Raffen R, Collart FR, Donnelly MI (2002). A new vector for high-throughput, ligation-independent cloning encoding a tobacco etch virus protease cleavage site.. Protein Expr Purif.

[25] Otwinowski Z, Minor W (1997). Processing of X-ray diffraction data collected in oscillation mode.. Method Enzymol.

[26] Sheldrick GM (2008). A short history of SHELX.. Acta Crystallogr A.

[27] McCoy AJ, Grosse-Kunstleve RW, Adams PD, Winn MD, Storoni LC (2007). Phaser crystallographic software.. J Appl Crystallogr.

[28] Collaborative Computational Project Number 4 (1994). The CCP4 suite: programs for protein crystallography.. Acta Crystallogr D Biol Crystallogr.

[29] Perrakis A, Harkiolaki M, Wilson KS, Lamzin VS (2001). ARP/wARP and molecular replacement.. Acta Crystallogr D Biol Crystallogr.

[30] Storoni LC, McCoy AJ, Read RJ (2004). Likelihood-enhanced fast rotation functions.. Acta Crystallogr D Biol Crystallogr.

[31] Murshudov GN, Vagin AA, Dodson EJ (1997). Refinement of macromolecular structures by the maximum-likelihood method.. Acta Crystallogr D Biol Crystallogr.

[32] Adams PD, Grosse-Kunstleve RW, Hung LW, Loerger TR, McCoy AJ, Moriarty NW, Read RJ, Sacchettini JC, Sauter NK, Terwilliger TC (2002). PHENIX: building new software for automated crystallographic structure determination.. Acta Crystallogr D Biol Crystallogr.

[33] Emsley P, Cowtan K (2004). Coot: model-building tools for molecular graphics.. Acta Crystallogr D Biol Crystallogr.

[34] Davis IWML, Richardson JS, Richardson DC (2004). MOLPROBITY: structure validation and all-atom contact analysis for nucleic acids and their complexes.. Nucleic Acids Res.

[35] Shenoy SR, Jayaram B (2010). Proteins: sequence to structure and function–current status.. Curr Protein Pept Sci.

[36] Zhang Y (2008). I-TASSER server for protein 3D structure prediction.. BMC Bioinformatics.

[37] Wu S, Zhang Y (2008). MUSTER: Improving protein sequence profile-profile alignments by using multiple sources of structure information.. Proteins.

[38] Kelley LA, Sternberg MJ (2009). Protein structure prediction on the Web: a case study using the Phyre server.. Nat Protoc.

[39] Wallner B, Fang H, Elofsson A (2003). Automatic consensus-based fold recognition using Pcons, ProQ, and Pmodeller.. Proteins.

[40] Chen CC, Hwang JK, Yang JM (2009). (PS)2-v2: template-based protein structure prediction server.. BMC Bioinformatics.

[41] Kim DE, Chivian D, Baker D (2004). Protein structure prediction and analysis using the Robetta server.. Nucleic Acids Research.

[42] Karplus K (2009). SAM-T08, HMM-based protein structure prediction.. Nucleic Acids Res.

